# Transmission of α-synuclein-containing erythrocyte-derived extracellular vesicles across the blood-brain barrier via adsorptive mediated transcytosis: another mechanism for initiation and progression of Parkinson’s disease?

**DOI:** 10.1186/s40478-017-0470-4

**Published:** 2017-09-13

**Authors:** Junichi Matsumoto, Tessandra Stewart, Lifu Sheng, Na Li, Kristin Bullock, Ning Song, Min Shi, William A Banks, Jing Zhang

**Affiliations:** 1 Department of Pathology, University of Washington School of Medicine, Seattle, WA 98104 USA; 2 Department of Pathology, Peking University Health Science Centers, Beijing, 100191 China; 3 Geriatrics Research Education and Clinical Center, Veterans Affairs Puget Sound Health Care System, Seattle, WA 98108 USA; 4 Division of Gerontology and Geriatric Medicine, Department of Medicine, University of Washington School of Medicine, Seattle, WA 98108 USA

**Keywords:** Extracellular vesicles, Blood-brain barrier, Alpha-synuclein, Parkinson’s disease, Inflammation, Microglia

## Abstract

Parkinson’s disease (PD) pathophysiology develops in part from the formation, transmission, and aggregation of toxic species of the protein α-synuclein (α-syn). Recent evidence suggests that extracellular vesicles (EVs) may play a vital role in the transport of toxic α-syn between brain regions. Moreover, increasing evidence has highlighted the participation of peripheral molecules, particularly inflammatory species, which may influence or exacerbate the development of PD-related changes to the central nervous system (CNS), although detailed characterization of these species remains to be completed. Despite these findings, little attention has been devoted to erythrocytes, which contain α-syn concentrations ~1000-fold higher than the cerebrospinal fluid, as a source of potentially pathogenic α-syn. Here, we demonstrate that erythrocytes produce α-syn-rich EVs, which can cross the BBB, particularly under inflammatory conditions provoked by peripheral administration of lipopolysaccharide. This transport likely occurs via adsorptive-mediated transcytosis, with EVs that transit the BBB co-localizing with brain microglia. Examination of microglial reactivity upon exposure to α-syn-containing erythrocyte EVs in vitro and in vivo revealed that uptake provoked an increase in microglial inflammatory responses. EVs derived from the erythrocytes of PD patients elicited stronger responses than did those of control subjects, suggesting that inherent characteristics of EVs arising in the periphery might contribute to, or even initiate, CNS α-syn-related pathology. These results provide new insight into the mechanisms by which the brain and periphery communicate throughout the process of synucleinopathy pathogenesis.

**Electronic supplementary material:**

The online version of this article (10.1186/s40478-017-0470-4) contains supplementary material, which is available to authorized users.

## Introduction

Parkinson’s disease (PD), a common neurodegenerative disorder, is characterized by neuronal death in multiple brain regions, e.g. the substantia nigra that primarily drives movement disorders seen in PD patients. α-Synuclein (α-syn) protein is implicated in the etiology of PD, not only because mutations and duplications/triplications in *SNCA*, the gene encoding α-syn, cause early onset familial PD, but also because the protein itself is a principle component of intraneuronal aggregations called Lewy bodies, the histopathological hallmark of PD [30]. α-Syn is secreted into extracellular spaces, and extracellular α-syn has been suggested to play a role in the progression of PD, particularly in the so-called “prion-like” spread of α-syn pathology throughout the brain.

Intriguingly, increasing evidence has suggested that not only central nervous system (CNS) α-syn, but also α-syn derived from the periphery, may play a role in PD pathogenesis. Several studies have demonstrated that retrograde transport may transmit pathologic α-syn from the gut to the brain [11, 23, 32], while intramuscular injection of α-syn can exacerbate pathology in transgenic α-syn mice, suggesting that multiple pathways exist for the transmission of α-syn from the periphery to the brain [46]. Importantly, a recent study demonstrated that gut microorganisms influence α-syn-related PD pathology in an animal model, and linked this peripheral factor to the activation of brain microglia [47], CNS immune cells that contribute to the inflammatory environment characteristic of the PD brain.

Despite these findings, little attention has been paid to whether α-syn in the blood may play a role in PD development, even after the discovery that plasma levels of α-syn exceed those of cerebrospinal fluid (CSF) by about 10-fold [24, 50]. The main source of α-syn in the blood is the erythrocyte, i.e. red blood cells (RBCs), which contain around 98% of all α-syn in whole blood [7, 50]. Normally, the transport of this blood α-syn is controlled by the blood-brain barrier (BBB), the dynamic barrier between the peripheral circulatory system and the CNS, comprised largely of brain microvascular endothelial cells (BMECs), which regulate molecular exchange between blood and brain to maintain homeostasis in the CNS [63]. BMECs exhibit tight regulation of intercellular trafficking as evidenced by low paracellular diffusion and the expression of a wide variety of selective transporters and receptors. However, we have reported that free α-syn protein is transported across the BBB bi-directionally, from blood-to-brain and in reverse [52]. The bacterial endotoxin lipopolysaccharide (LPS) has been the most extensively utilized glial activator for the induction of inflammatory DA neurodegeneration [16]. We previously observed that lipopolysaccharide (LPS)-induced inflammation enhanced the blood-to-brain uptake of α-syn by disruption of BBB [52], a particularly interesting finding in light of emerging evidence suggesting that inflammation plays an important role in pathological features in PD [38].

More recently, we have found that α-syn can be transported from the brain to the blood within extracellular vesicles (EVs) [49]. EVs such as exosomes and microvesicles are small membrane-bound vesicles released by donor cells that can participate in the para-cellular transport of cargo proteins, RNAs, DNAs, and lipids from donor cells to recipient cells [28, 42], and have drawn attention as being important players in a variety of neurodegenerative diseases including PD. In the context of PD, some evidence suggests a role for exosome transport in the progression of PD via prion-like spread of pathogenic misfolded α-syn [1]. It was not previously known, however, whether α-syn might be transported within EVs in the opposite direction, from blood to brain. Further, the consequences of such transport, particularly under inflammatory conditions, on brain cells such as microglia, are unexplored, despite a previous study demonstrating that α-syn-carrying exosomes from other sources provoke microglial activation [9].

In this study, we aimed to characterize EVs released from RBCs, quantify their α-syn content, and examine their transport across the BBB, from the peripheral circulatory system to the CNS under LPS-induced systemic inflammation. We now show that LPS-induced systemic inflammation facilitates transfer of RBC-derived EVs containing α-syn via adsorptive-mediated transcytosis. RBC-derived EVs were observed in cerebral nuclei, cortex, interbrain, midbrain and substantia nigra, and co-localized with microglia. Erythrocyte-derived EVs from PD patients provoked a greater increase in microglial inflammatory responses than did EVs from healthy controls. These results suggest that peripheral insults that provoke systemic inflammation (i.e., sepsis or tissue trauma), can increase the transfer of RBC-derived α-syn into the brain, with profound, potentially long-term consequences for brain inflammation and function and, ultimately, the development of PD.

## Materials and Methods

### Animals and antibodies

All applicable international, national, and/or institutional guidelines for the care and use of animals were followed, and all procedures were approved by the Institutional Animal Care and Use Committees at the University of Washington or the VA Puget Sound Health Care Center. Eight week old male CD-1 mice were purchased from Charles River and kept on a 12/12-h light/dark cycle with ad libitum food and water. CD-1 mice are an outbred strain for use for genetics, toxicology, pharmacology, and aging research. In this study, a total of 166 mice were used in six different in vivo experiments: 14 for the clearance of EVs from blood, 30 for the uptake of RBC-EVs by brain, 30 for capillary depletion, 56 to test saturation using unlabeled RBC-EVs, 22 for the effect of WGA on permeability of RBC-EVs and 14 for immunofluorescence staining, respectively.

Mouse monoclonal antibodies against human Alix (2171S, Cell Signaling Technology, Danvers, USA) were used in Western blot (WB); mouse monoclonal antibodies against CD235a (MA5-12484, Thermo Fisher Scientific, Waltham, MA, USA) were used in WB; rabbit polyclonal antibodies against induced nitric oxide synthase (iNOS) (ab15323, Abcam, USA) were used in WB and IF; chicken polyclonal antibodies (ab139590, Abcam, USA) and rabbit polyclonal antibodies (019–19,741, Wako, Japan) against Iba-1 were used in IF; chicken polyclonal antibodies against GFAP (Thermo Fisher Scientific) were used in IF; mouse polyclonal antibodies against GAPDH (HC301, TRANS, China) were used in WB. Horseradish peroxidase (HRP)-conjugated secondary antibodies used in WB and Alexa Fluor 405, 488, 568 or 633 conjugated secondary antibodies used in IF were purchased from Thermo Fisher Scientific.

### Isolation of RBC-EVs from PD and normal human erythrocytes

All procedures performed in studies involving human participants were in accordance with the ethical standards of the institutional and/or national research committee and with the 1964 Helsinki declaration and its later amendments or comparable ethical standards. Approval was granted by the Institutional Review Board at Peking University Health Science Center, and all subjects were fully informed and consented to the study. Human erythrocytes from healthy control subjects (ZenBio, Research Triangle Park, North Carolina), Parkinson’s disease (PD) patients, or age-matched controls (Additional file 1: Table S1), were first subjected to a RPMI-1640 culture medium containing 25 mM HEPES. Cells were maintained at 37 °C in a CO_2_ incubator for 48 h. RBC-EVs were then isolated from culture medium via serial centrifugations. Culture medium was collected and centrifuged at 1500×g for 10 min, followed by centrifugation at 3000×g for 15 min (two times) to remove the intact cells, cell debris and apoptotic blebs. EVs were concentrated from the supernatants by centrifugation at 150,000×g for 2 h, the resulting pellet washed with PBS, and collected by centrifugation at 200,000×g for 2 h. Sepharose CL-2B (Sigma, St Louis, MO, USA) was found to be suitable for separating EVs from small molecular proteins such as albumin [10, 57]. The EV sample was purified using CL-2B column. Each elution (0.5 mL) was collected to a 1.5 mL tube and the protein concentration of each fraction was measured based on UV absorbance at 280 nm by NanoDrop™ Lite Spectrophotometer (Thermo fisher Scientific). Two different observed peaks (Peak 1 and Peak 2) were further concentrated using Amicon® Ultra centrifugal filter devices (cut-off MW 100 kDa, Millipore Corporation, Billerica, MA, USA).

### Nanoparticle tracking analysis (NTA)

The number of particles and size distribution in each peak were analyzed with NTA (NS300; Nanosight, Amesbury, UK). Each fraction was diluted 1000-fold in PBS in order to optimize the number of particles in each fraction. For each fraction, three videos (60 s each) were captured, and then all fractions were analyzed using the same threshold. Analysis was performed by NTA 3.1 software (Nanosight, Amesbury, UK).

### Luminex immunoassays

RBC-EVs or RBC cell lysates were used to quantify α-syn with an established Luminex protocol as previously described [24]. Briefly, the Luminex assay was performed in 96 well MultiScreen Filter plates (Millipore) in the dark at room temperature. The plate was pre-wetted with a working solution (Na_2_HPO_4_ 1.175 mg/mL, NaH_2_PO_4_ 0.228 mg/mL NaCl 8.77 mg/mL, NaN_3_ 0.05 mg/mL 0.2% Tween, 0.2% TrironX-100, pH 7.4). The capturing antibody (anti-α-syn antibody ASY-1, a gift from Dr. Poul H. Jensen, University of Aarhus, Denmark)-coupled beads were mixed thoroughly by vortexing (30 s) and brief sonication (30 s) and were immediately added to the pre-wetted plate. 5 μg of RBC-EVs or RBC cell lysates were added into each well (100 μl/well) and incubated for 3 h at 600 rpm on a plate shaker. After incubation, the sample solution was removed, and then the plate was washed three times by working solution. The detecting antibodies in assay diluent [0.1% Bovine Serum Albumin (BSA) in PBS] [1 ng/ml; biotinylated anti-human α-synuclein antibody (R&D systems, Minneapolis, MN, USA)] were added at 100 μl /well and incubated for 3 h on a plate shaker (600 rpm). After the detecting antibody solution was removed, the plate was washed three times with washing solution. A streptavidin-RPE (1 ng/ml; Prozyme, St. Louis, MO, USA) in assay diluent was added (100 μl /well) and incubated for 30 min on a plate shaker (600 rpm). The plate was then washed four times and 100 μl of washing solution was added to each well. After incubation for 5 min on a plate shaker, the plate was read on a LiquiChip Luminex 200TM Workstation (Qiagen, Valencia, CA, USA). A calibration curve consisting of a series of recombinant full-length human α-synuclein (rPeptide, Athens, GA, USA) standards diluted in assay diluent solution were run in parallel.

### Radioactive labeling of RBC-EVs and albumin

RBC-EVs were radioactively labeled with Na^125^I or Na^131^I (Perkin Elmer, Waltham, MA, USA) by chloramine-T (Sigma Aldrich) as previously described [11]. Radioactively labeled RBC-EVs were purified on an Illustra NAP5 column (GE Healthcare, Buckinghamshire, UK). Albumin (Sigma Aldrich) was radioactively labeled with ^99m^Tc. Radioactively labeled albumin (^99m^Tc-Alb) was purified on a Sephadex G-10 column (Sigma Aldrich). The radiolabeling efficiency was determined by trichloroacetic acid precipitation method. 1 μl of sample was added into 500 μl of Lactated Ringer solution containing 1% BSA (LR-BSA) and mixed with an equal volume of 30% TCA, and then centrifuged at 5400 × g for 10 min. The levels of radioactivity in the pellet and the supernatant were separately measured in a gamma counter for 3 min (PerkinElmer). The radiolabeling efficiency was calculated as the percentage of pellet radioactivity to total (pellet plus supernatant) radioactivity. The radiolabeling efficiency for RBC-EVs and Albumin was about 80 and 90%, respectively, across different preparations.

To assess the stability of radiolabeled RBC-EVs, samples loaded into mouse serum were incubated for 0, 15 and 60 min. These samples were mixed with 30% TCA and then centrifuged at 5400 × g for 10 min. The levels of radioactivity in the pellet and the supernatant were separately measured in a gamma counter for 3 min (PerkinElmer). The radiolabeling efficiency was calculated as the percentage of pellet radioactivity to total radioactivity. Then, the stability or radiolabeled RBC-EVs were calculated as the percentage of radiolabeling efficiency at 15 min or 60 min to radiolabeling efficiency at 0 min. The stability of radiolabeled RBC-EVs at 15 min and 60 min was about 98 and 90%, respectively.

### In vivo evaluation of RBC-EV permeability

Mice were weighed and given an intraperitoneal (IP) injection of LPS (3 mg/kg dissolved in sterile normal saline) from Salmonella typhimurium (Sigma Aldrich) or control saline three times (at 0, 6 and 24 h). Mice underwent the experiment 28 h after the first injection of LPS or control saline. Multiple-time regression analysis [8, 34] was used to calculate blood-to-tissue (brain, liver, spleen and kidney) uptake of ^125^I-EVs and Tc^99^m-Alb. In vivo evaluation of RBC-EVs was carried out according to the methods described previously [4, 6]. CD-1 mice were anesthetized with 40% urethane and a 200 μL injection of Lactated Ringer solution containing 1% BSA (LR-BSA), 300,000 cpm of ^125^I-RBC-EVs and Tc^99^m-Alb each was injected into the jugular vein (IV injection). Between 1 to 15 min after the IV injection, the arterial blood was collected from the carotid artery. The collected whole blood was centrifuged at 3000×g for 15 min, and 50 μL of serum transferred into a new glass tube. Levels of radioactivity in 50 μL of serum were measured in a gamma counter for 3 min. The tissue was removed after blood collection at each time-point and weighed. The levels of radioactivity in the tissue were determined in a gamma counter. The results were expressed as tissue (brain, liver, kidney and spleen)/serum ratios in units of μL/g and plotted against exposure time (expt) in units of minutes. The slope of the line for the linear portion of the relation between tissue/serum ratios and expt measures the unidirectional influx rate (Ki) in units of μL/g-min and the intercept of that line measures the initial volume of distribution in tissue (Vi) in units of μL/g. The half-time clearance and initial volume of distribution in the body (Vd) were calculated according to the method as previously described [5].

For self-inhibition studies, 300,000 cpm of ^125^I-RBC-EVs with or without 1 or 30 μg of unlabeled RBC-EVs was injected via jugular vein and mice were decapitated 15 min after IV injection and brain and serum collected.

To examine the effect of wheat germ agglutinin (WGA) (Sigma Aldrich) on the permeability of RBC-EVs, ^131^I-RBC-EVs with or without 10 μg per mouse of WGA was injected via jugular vein and mice were decapitated 15 min after IV injection and collect brain and serum.

Capillary depletion was carried out according to the methods described previously [6]. Mice were decapitated 15 min after IV injection and brain and serum collected. The collected mouse brain was weighed and homogenized with 0.8 mL of physiological buffer (10 mM HEPES, 141 mM NaCl, 4 mM KCl, 2.8 mM CaCl_2_, 1 mM MgSO_4_, NaH_2_PO_4_ and 10 mM d-glucose adjusted pH 7.4) at 4 °C. 1.6 mL of 26% dextran solution in physiological buffer added to the brain homogenate. The pellet containing the brain capillary was carefully separated from the supernatant containing the brain parenchyma after centrifuge at 5400×g for 15 min at 4 °C. The levels of radioactivity in capillary or brain parenchyma were determined in a gamma counter. The results were expressed as capillary/serum ratios and brain parenchyma/serum in units of μL/g.

### Culture of primary BMECs

Primary BMECs were isolated from 8-week-old CD-1 mice as previously described [2]. Briefly, isolated BMECs were cultured using BMEC medium, which contained Dulbecco’s modified Eagle’s medium (DMEM)/F12 supplemented with 20% plasma-derived fetal bovine serum (Animal Technologies), 1% GlutaMAX (Thermo fisher Scientific), basic fibroblast growth factor (bFGF, 1 ng/ml; Roche Life Sciences), heparin (100 μg/ml), insulin (5 μg/ml), transferrin (5 μg/ml), selenium (5 ng/ml) (Thermo fisher Scientific), and gentamicin (50 μg/ml) (Sigma Aldrich). Puromycin (4 μg/ml) (Sigma Aldrich) for the first 48 h after plating. Cells were maintained at 37 °C in a CO_2_ incubator for 24 h. Culture medium was changed at 24 h after plating to remove non-adhering cells, RBCs, and debris. At 48 h after plating, the medium was changed to BMEC medium in the absence of puromycin. The purified primary BMECs were used to construct in vitro models when reached 80% confluency.

### Construction of in vitro BBB models

In vitro BBB models were constructed as previously described [2]. Briefly, BMECs (4 × 10^4^ cells/ well) were seeded onto fibronectin and collagen type IV pre-coated (0.1 mg/ml, each) Transwell inserts (0.33 cm^2^, 0.4-μm pore size, Corning). For the negative controls, no BMECs were seeded to the collagen type IV plus fibronectin pre-coated Transwell insert. BMEC medium containing additional hydrocortisone (500 nM) was used to reinforce tight junctions of in vitro BBB models [22]. An EVOM volt ohmmeter connecting with a STX-2 electrode (World Precision Instruments; Sarasota, FL) was used to analyze of transendothelial electrical resistance (TEER, in ohms per square centimeter) prior to treatment. The TEER value of cell-free Transwell inserts (negative controls) was subtracted from TEER value of BMECs containing Transwell inserts. BMEC monolayers were established within 7 days after seeding in Transwell inserts.

### Transendothelial permeability assay

A permeability assay for RBC-EVs was conducted according to the method described previously [2]. A 600 μL volume of DMEM/F12 containing 1% BSA (an assay buffer) was added to the wells of a new 24-well plate, and the inserts were put on in these wells. ^125^I-RBC-EVs (3 × 10^6^ cpm/mL) in the assay buffer (100 μL) were added to the luminal chamber (loading chamber). Samples (400 uL) were mixed with pipetting and collected from the abluminal chamber (collecting chamber) to a new glass tube at 15, 30, 45, 60 min, and added an equal volume of fresh DMEM/F12 containing 1% BSA to the abluminal chamber after sample collection at each time point. 400 uL of 30% trichloroacetic acid (TCA) added to all samples and centrifuged at 5400×g for 15 min at 4 °C. After removal of the supernatant, the radioactivity in the TCA precipitate was determined in a gamma counter. The permeability coefficient of TCA-precipitable ^125^I-RBC-EVs (cm/min) was calculated from the clearance of TCA-precipitable ^125^I-RBC-EVs (μL) according to the method described previously [2]. Clearance was expressed as microliters of radioactive tracer diffusing from the luminal chamber to the abluminal chamber and was calculated from the initial level of radioactivity in the luminal chamber (loading chamber) and final level of radioactivity in the abluminal chamber (collecting chamber):

$$ \mathrm{Clearance}\ \left(\upmu \mathrm{L}\right)={\left[\mathrm{C}\right]}_{\mathrm{C}}\times {V}_{\mathrm{C}}/{\left[\mathrm{C}\right]}_{\mathrm{L}}, $$

where [C]_L_ is the initial radioactivity in a microliter of loading chamber (in cpm/μL), [C]_C_ is the radioactivity in a microliter of collecting chamber (in cpm/μL), and *V*_C_ is the volume of collecting chamber (in μL). During a 60-min period of the experiment, the clearance volume increased linearly with time. The volume cleared was plotted versus time, and the slope was estimated by linear regression analysis. The slope of clearance curves for the BMEC monolayer plus Transwell® membrane was denoted by *PS*_app_, where *PS* is the permeability × surface area product (in μL/min). The slope of the clearance curve with a Transwell® membrane without BMECs was denoted by *PS*_membrane_. The real *PS* value for the BMEC monolayer (*PS*_e_) was calculated from 1/*PS*_app_ = 1/*PS*_membrane_ + 1/*PS*_e_. The *PS*_e_ values were divided by the surface area of the Transwell® inserts (0.33 cm^2^) to generate the endothelial permeability coefficient (*P*_e_, in cm/min).

### N9 Microglia culture and stimulation by RBC-derived EVs

Mouse N9 cell line (N9 microglia), a retroviral-immortalized cell line (kindly provided by Department of Pathology, Peking University Health Science Centers), were plated in 6-well plate at a density of 5 × 10^5^ cells/well with F12/DMEM containing 10% FBS and incubated at 37 °C in a CO_2_ incubator overnight, then the medium was replaced with F12/DMEM free of FBS. Simultaneously, 100 μl dissolved RBC-EVs derived from PD patients or control subjects was added and cells were stimulated for 30 min.

### Western blot analysis

RBC-EVs and RBC cell lysates were mixed with an equal volume of 2 × Laemmli sample buffer (Bio-Rad Laboratories, Hercules, CA, USA). Equivalent amounts of protein from each sample were electrophoretically separated on 4-15% Criterion™ TGX Stain-Free™ Protein Gel (Bio-Rad Laboratories) and then transferred to polyvinylidene difluoride (PVDF) membranes (Bio-Rad Laboratories). Membranes were blocked with Blocking One (Nacalai Tesque, Kyoto, Japan). The membrane was probed with corresponding primary antibodies overnight at 4 °C. After washing, membranes were then incubated with appropriate horseradish peroxidase (HRP)-conjugated secondary antibodies. The immunoreactive bands were visualized using ECL reagents (Amersham Pharmacia Biotech, Buckinghamshire, UK).

Proteins were extracted from N9 microglia by cell lysis buffer (RIPA cell lysis buffer, PPLYGEN, C1053) and the protein concentration was determined by BCA Protein Assay Kit according to the manufacturer’s instruction. The sample was boiled in 5 × SDS loading buffer for 5 min and loaded onto a 10% SDS-polyacrylamide gel. Following electrophoresis, the proteins were transferred to a PVDF membrane (Merck Millipore). The membranes were blocked for 1 h at RT in 5% BSA (amresco) in TBST buffer (Axygen). Immunoblotting was performed by incubating the membrane in 5% BSA-TBST with corresponding primary antibodies overnight at 4 °C. The membranes were washed three times with TBST, followed by incubation with appropriate HRP-conjugated secondary antibodies, positive bands were detected using enhanced chemiluminescence reagents (Millipore) and quantified using densitometric analyses by Photoshop.

### Immunofluorescence staining

The RBC-EVs were labeled with Vybrant™ DiI cell-labeling solution in accordance with the manufacturer’s instructions (Thermo Fisher Scientific). Briefly, RBC-EVs were resuspended in 500 μL of PBS. DiI solution (10^−3^ μmol) was then added into resuspended RBC-EVs in PBS, followed by 20 min incubation at room temperature. To remove the excess DiI dye, DiI-labeled RBC-EVs were further concentrated using Amicon® Ultra centrifugal filter devices (cut-off MW 100 kDa), and then re-suspended with PBS three times and finally resuspended in 50 μL of PBS.

Mice were anesthetized with i.p. injection of 0.15 ml of 40% urethane (Sigma Aldrich) [52]. DiI-labeled RBC-EVs dissolved in PBS (50 μg per mice) or control PBS were intravenously injected via the jugular vein to LPS (3 mg/kg of mice) or control saline pre-injected (i.p. injection) mice. After 3 h, the mice were perfused. The descending aorta was clamped and both jugular veins were severed. A 23-gauge butterfly needle was injected into the left ventricle of the heart and then PBS infused at a rate of 2 ml/min for 5 min, followed by perfusion of 4% paraformaldehyde solution at a rate of 2 ml/min for 5 min. Brains were removed and immersed in 4% paraformaldehyde solution at 4 °C overnight. After dehydration in 20% sucrose, sagittal brain sections (20 μm) were prepared with a sliding microtome (Leica, Wetzlar, Germany).

Brain slices were washed with PBS and treated with blocking solution (1% BSA, 0.4% Triton X-100 and 4% goat serum in PBS) for 2 h. Next, brain slices were incubated overnight at 4 °C with primary antibodies diluted in blocking solution. Brain slices were then washed with washing buffer (0.1% Tween in PBS) and incubated with corresponding secondary antibodies diluted in PBS containing 0.3% of Triton X-100 for 2 h. After washing with PBS or washing buffer and PBS (when indicated), brain slides were embedded in Vectashield medium or Vectashield medium with DAPI (when indicated).

Immunofluorescence images were captured at room temperature using a Nikon Eclipse Ti (Nikon Instruments Inc., Melville, NY, USA) instrument under 20× or 40× magnification. Z-series images were acquired from randomly selected presence or absence DiI-labeled RBC-EVs fields, following with deconvolution. Interbrain including thalamus and hypothalamus were analyzed. Midbrain including Substantia nigra was analyzed. Isocortex of cerebral cortex was analyzed. Note that DiI signal in the brain may signify that EV membrane is present but cannot inform on the number of EVs or their cargo.

### Statistical analysis

The results are shown as means ± S.E.M. The statistical significance of differences between two groups was assessed by the Student’s t-test, one-way or two-way analysis of variance (ANOVA), followed by Tukey-Kramer’s post-hoc test for multiple comparisons and Kruskal-Wallis test, followed by Dunn’s post-hoc test for multiple comparisons (Graph Pad Prism 5.0 (GraphPad, San Diego, CA)). *p < 0.05; **p < 0.01; ***p < 0.001.

## Results

### EVs of RBC contain α-synuclein

**Fig. 1:**
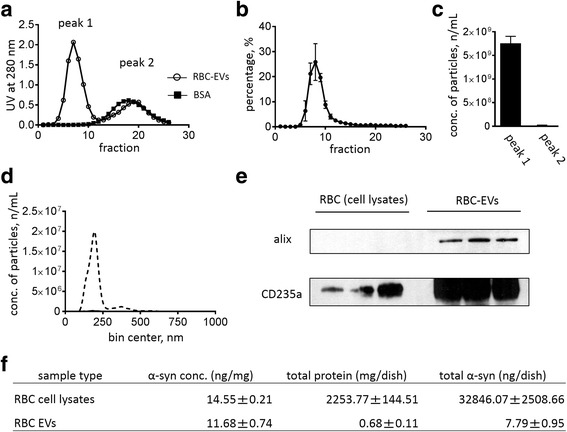
The characterization of extracellular vesicles from packed red blood cells (RBC-EVs). SEC was used to separate EVs (peak 1) from low molecular weight protein contaminants [peak 2; co-eluted with BSA (66 kDa)]. The presence of (EV surface) proteins was monitored by UV absorbance at 280 nm (**a**), and the number of particles in each fraction was further measured by NTA (**b**). The total particle number of RBC-EVs (**c**) and the distribution of RBC-EVs (**d**) were measured in each peak by NTA (Nanosight NC300). Representative images of Western blots showing Alix and CD235a in RBC-EVs and RBC cell lysate **e** Three independently cultured and prepared samples of RBC lysate and EVs (50 μg of total proteins in each sample) were used for western blot analysis and were tested by immunoblot with antibodies against Alix (exosome marker) and CD235a (RBC marker). The α-syn level in RBC-EVs was measured using Luminex immunoassay (**f**), compared to the α-syn level in RBC cell lysates. Values are means ± S.E.M, *n* = 3

Size exclusion column (SEC) and NTA were used to characterize RBC-EVs. NTA uses Brownian motion and light scattering to determine the size distribution of particles in a sample. We use a Nanosight instrument, which enables characterization of particles from 10 to 1000 nm in solution [58]. As shown in Fig. 1a, there are two protein peaks (UV absorption of 280 nm) in CL-2B SEC analysis. The first peak (peak 1; EV fraction) was observed from Fraction 6 to Fraction 8 and the second (peak 2; low molecular weight protein fraction) was observed from Fraction 15 to Fraction 22, which also co-eluted with BSA (66 kDa) (Fig. 1a). We confirmed that EVs were eluted from fraction 6 to fraction 10 subjecting each fraction to NTA and quantifying particle number (Fig. 1b). After collection of each fraction, peak 1 and peak 2 were concentrated by centrifugation using a 100 kDa cut-off filter. NTA analysis showed that the total number of nanoparticles in peak 1 was about 95-fold higher than peak 2 (peak1: 1.76 × 10^9^ particles/mL, peak 2: 1.84 × 10^7^ particles/mL) (Fig. 1c). The average size of nanoparticles in peak 1 was 205.22 ± 1.79 nm (Fig. 1d).

Western blot analysis demonstrated that Alix, a general exosome protein, was detected in the EVs, but not in RBC cell lysates where its level was below the detection limit of the western when an equal amount (50 μg) of total proteins was loaded. In contrast, CD235a, an RBC marker, was detected in RBC-EVs and RBC cell lysates (Fig. 1e). We used our previously established bead-based Luminex [24] immunoassay to measure α-syn in RBC-EVs and RBC cell lysates. The Luminex assay showed that, after correction for protein loaded, the α-syn concentration of RBC-EVs is comparable to that of RBC cell lysate (RBC-EVs 11.68 ± 0.74 ng/mg, RBC cell lysate 14.55 ± 0.21 ng/mg) (Fig. 1f). The protein in RBC-EVs makes up only 0.03% of total protein amount of RBC cell lysates, so total α-syn amount was much greater in RBC cell lysates than in RBC-EVs (RBC-EVs 7.79 ± 0.95 ng/dish, RBC cell lysate 34,846.07 ± 2508.66 ng/dish).

### Lipopolysaccharide facilitated cross of BBB by EVs

**Fig. 2:**
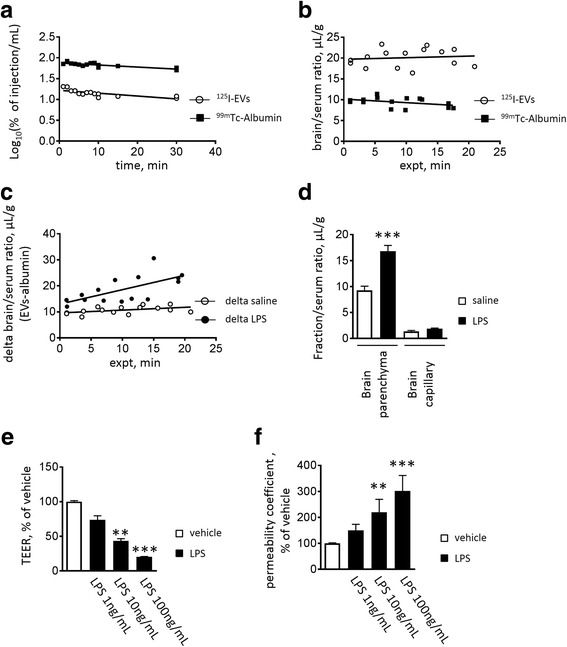
RBC-EVs cross the BBB after 3 injections of LPS: Clearance of intravenously injected ^125^I-EVs from blood. Pharmacokinetic parameters were calculated for the relation between log10 (%Inj/mL) and time **a** For ^125^I-EVs, calculations based on the slope yielded a half-time clearance of 44 min and based on the Y-intercept, yielded an initial volume of distribution (Vd) of 6.02 mL. For Tc^99^m-albumin, half-time clearance was 61 min and an initial Vd was 1.32 mL. Uptake of ^125^I-EVs and Tc^99^m-Alb by brain **b** Unidirectional influx rates (μL/g-min) for brain were calculated by multiple-time regression analysis, *n* = 15/group. Unidirectional influx rates of ^125^I-EVs and Tc^99^m-Alb by brain following LPS **c** Multiple-time regression analysis was used to calculate the unidirectional influx rates of delta values (brain serum ratios for ^125^I-EVs with brain/serum ratios for Tc^99m^-Alb subtracted) vs exposure time. LPS significantly increased the uptake by brain of ^125^I-EVs (*p* < 0.05), *n* = 15/group. Capillary depletion with or without LPS **d** The majority of RBC-EVs was in the parenchymal space. LPS significantly increased the crossing of ^131^I-EVs in brain parenchyma. Values are means ± S.E.M. Student's t-test *** p < 0.001, *n* = 7-8/group. LPS dose-dependently decrease TEER (**e**) and increase the permeability of ^125^I-EVs **f** Results are expressed as % of vehicle (74.6 ± 11.9 Ω cm^2^ (**e**); 2.7 ± 0.4 × 10^−5^ cm/min (**f**)). Values are means ± S.E.M. Kruskal-Wallis test ** p < 0.01, *** p < 0.001 vs vehicle, *n* = 8–9

To assess the behavior of RBC-EVs in the whole body, as well as whether they enter the brain, we performed a pharmacokinetic (PK) study. RBC-EVs were treated with ^125^I, resulting in labeling of all external proteins on the EV membrane surface. First, we evaluated the clearance of ^125^I-RBC-EVs and ^99m^TC-Alb (vascular marker) from peripheral circulation, by injecting the labeled EVs peripherally and observing remaining signal over time. Fig. 2a shows that ^125^I-RBC-EVs and ^99m^Tc-Alb cleared gradually from blood after iv injection. The half-time clearance for ^125^I-RBC-EVs and ^99m^TC-Alb was 44 min and 61 min, respectively. The volume of distribution, calculated from the slope and y-intercept, for ^125^I-RBC-EVs and ^99m^TC-Alb was 6.02 mL and 1.32 mL, respectively, suggesting a greater amount of EVs distributed into body tissue rather than the plasma compared to Alb. Next, we measured the unidirectional influx constant (Ki) of ^125^I-RBC-EVs into brain by multiple-time regression analysis in order to evaluate the transport of ^125^I-RBC-EVs from the peripheral blood to the brain. As shown in Fig. 2b ^125^I-RBC-EVs crossed the BBB from blood to the brain with a Ki of 0.03822 ± 0.0954 μL/g-min (Fig. 2b). ^99m^Tc-Alb was used as a BBB impenetrable marker and as a measure of vascular space. The slope of ^125^I-RBC-EVs did not show a significant difference when compared to the slope of ^99m^Tc-Alb (Ki = −0.08511 ± 0.05234 μL/g-min). The levels of ^125^I-RBC-EV uptake were higher in liver, kidney, and spleen than brain (Additional file 2: Figure S1). Consistent with previous PK studies [26, 57], EV transfer from the peripheral circulatory system to the CNS is rare under physiological conditions, with the EVs instead accumulating in liver, kidney and spleen.

To determine whether LPS induced ^125^I-RBC-EV permeability of the BBB, we injected LR buffer containing ^125^I-RBC-EVs and ^99m^Tc-Alb in mice pre-treated with three injections of LPS. The Ki of mice with LPS pretreatment was significantly higher than that of control mice, suggesting that ^125^I-RBC-EVs crossed the BBB more easily (Fig. 2c; control; 0.1079 ± 0.05487 μL/g-min LPS; 0.5533 ± 0.1704 μL/g-min, *p* = 0.0199). To demonstrate that ^125^I-RBC-EVs pass the BBB to enter the parenchyma, rather than accumulating in brain capillaries, we did a capillary depletion experiment which measured separately the radioactivity of brain parenchyma and that of brain capillary. This experiment showed that the majority of ^131^I-RBC-EVs can enter the parenchymal space of the brain and LPS increased crossing of ^131^I-RBC-EVs at the BBB (Fig. 2d). Finally, we examined whether LPS directly affects the in vitro luminal-to-abluminal influx of ^125^I-RBC-EVs across the BMEC monolayer (in vitro BBB model). As shown in Fig. 2e, we found that adding LPS to the luminal side resulted in a concentration-dependent decrease in TEER (an indicator for integrity of tight junctions at the BBB) in these cells, indicating higher permeability. We also showed that LPS significantly increased the number of ^125^I-RBC-EVs crossing BMEC monolayer.

### Adsorptive mediated transcytosis in lipopolysaccharide-induced EVs entry to brain

**Fig. 3:**
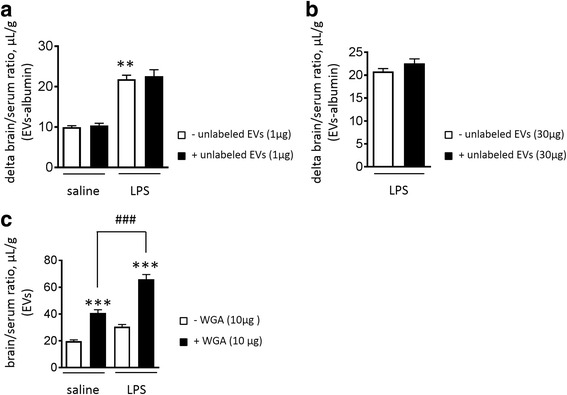
The effect of unlabeled EVs or WGA on ^125^I-EVs unidirectional influx following LPS. Both low (1 μg/mouse)- (**a**) and high (30 μg/mouse)- dose (**b**) of unlabeled EVs did not inhibit the increase of ^125^I-EVs uptake by brain in LPS treated mice. Values are means ± S.E.M. Kruskal-Wallis test ** p < 0.01 vs saline without unlabeled RBC-EVs *n* = 9-10/group. The effect WGA on the uptake by brain of ^131^I-EVs **c** WGA increased the uptake by brain of ^131^I-EVs in both saline and LPS treated mouse. Values are means ± S.E.M. One way ANOVA*** p < 0.001 vs saline without WGA or LPS without WGA, *### p* < 0.001 vs saline with WGA, *n* = 5-6/group

As shown in Fig. 3a, co-injection of either 1 μg/mouse or 30 μg/mouse dose of unlabeled ^125^I-RBC-EVs did not affect LPS-induced entry of ^125^I-RBC-EVs into the brain (Fig. 3a and b), suggesting that the mechanism of RBC-EVs transfer across the BBB did not occur via a saturable transport process. This led us to hypothesize that RBC-EVs can cross BBB via adsorptive-mediated transcytosis (AMT). Cationic proteins such as wheat germ agglutinin (WGA) can cross the BBB via AMT [59]. WGA is a glycoprotein lectin and a potent inducer of AMT in brain endothelial cells [3]. We next tested the effect of WGA, which increases uptake via AMT, on the ^131^I-RBC-EV permeability of the BBB in LPS-injected mice. WGA alone increased ^131^I-RBC-EVs uptake when compared to controls. Interestingly, our observation indicated that the value for mice receiving LPS in the presence of WGA was much higher than the value for mice receiving saline in the presence of WGA (Fig. 3c; *p* < 0.001).

### RBC-EVs were co-localized with microglia in LPS-injected mouse brains

**Fig. 4:**
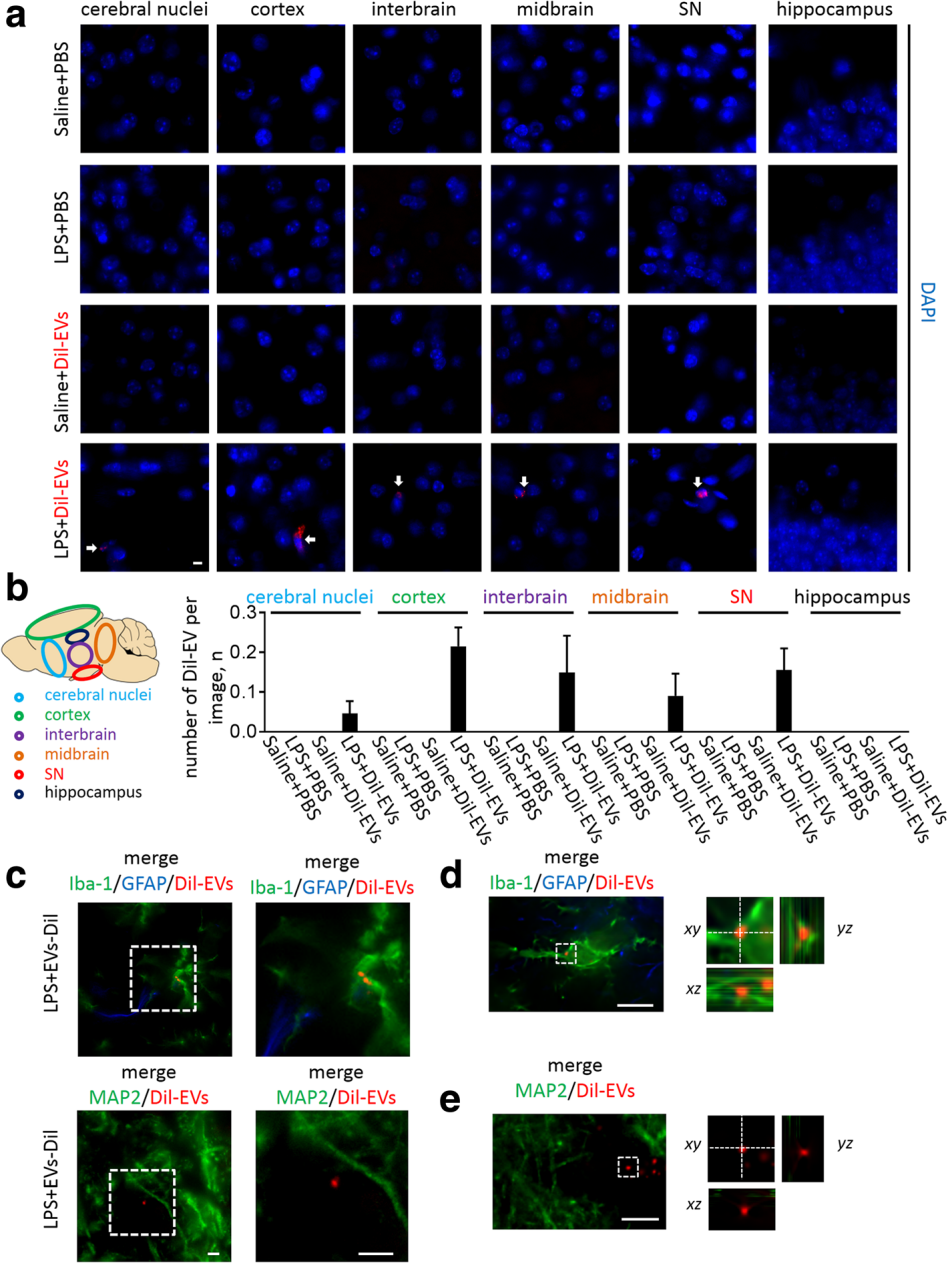
Peripheral injection of LPS promotes the influx of RBC-EVs crossing BBB and uptake of RBC-EVs by microglia. Images of brain slice with peripheral injection of LPS + DiI-EVs, LPS + PBS, saline + DiI-EVs or saline + PBS were labeled with DAPI to visualize the cells **a** Note that DiI-EVs can be only detected in LPS + DiI-EVs injected group at cerebral nuclei, cortex, interbrain, midbrain, substantia nigra (SN), but not at hippocampus. Scale bar, 10 μm. Graphs show numbers of DiI-EVs co-localized with DAPI in cerebral nuclei, cortex, interbrain, midbrain, substantia nigra and hippocampus in mouse with LPS + DiI-EVs, LPS + PBS, saline + DiI-EVs or saline + PBS administration **b** *n* = 3 independent animals in each group. Images of brain slice were co-labeled with Iba-1 and GFAP or labeled with MAP2 **c** Note that DiI-EVs co-localized with Iba-1. Scale bar, 10 μm. Images of brain slice were co-labeled with Iba-1 and GFAP **d** or labeled with MAP2 **e** Three-dimensional analysis of the co-localization within the outlined area is on the right. The *xz* and *yz* sections along the dashed lines on the *xy* image are shown. Note co-localization of the DiI-EVs with Iba-1 labeled microglia

To further confirm whether RBC-EVs can be transported into CNS, we next used a lipophilic fluorescence dye to label and track EVs in CNS. Brain slices from mice injected with LPS and DiI-labeled EVs, saline and DiI-labeled EVs, LPS or saline were fixed and labeled with DAPI to visualize cells. Immunofluorescence analysis of brain slices showed that fluorescence signals of DiI-labeled EVs were detected at a low rate in LPS-DiI-labeled EVs injected mice, while no fluorescence signals of DiI-labeled EVs were detected in saline-DiI-labeled EVs, LPS only or saline injected mice (Fig. 4a). Brain slices of LPS and DiI-dye or saline and DiI-dye injected mice did not show the labeling of DiI-labeled EVs (results not shown). We next analyzed what regions of the brain contain RBC-EVs. Interestingly, RBC-EVs were counted in cerebral nuclei, cortex, interbrain, midbrain and substantia nigra but not in the hippocampus of LPS injected mice (Fig. 4a and b).

To investigate which cell types can take up RBC-EVs, we labeled brain slices with antibodies against GFAP, an astrocytic marker protein, MAP2, a neuronal marker protein, or Iba-1, a microglial marker protein that shows better specificity in targeting microglia (rather than macrophages) compared to other commonly used markers [20]. Interestingly, RBC-EVs were visualized in ~1% of cells stained for the microglial marker protein Iba-1 in LPS injected mice (Fig. 4c and d). All Iba-1 positive cells identified had microglial morphology and were located within the brain parenchyma, not the capillaries, suggesting their likely microglial identity. No RBC-EVs were co-localized with GFAP-labeled astrocytes or MAP2-labeled neurons (Fig. 4d and e). Taken together, our observations suggest that RBC-EVs can be transported into CNS in LPS-injected mice and can be taken up by microglia.

### PD-derived RBC-EVs increased the pro-inflammatory properties in microglia

**Fig. 5:**
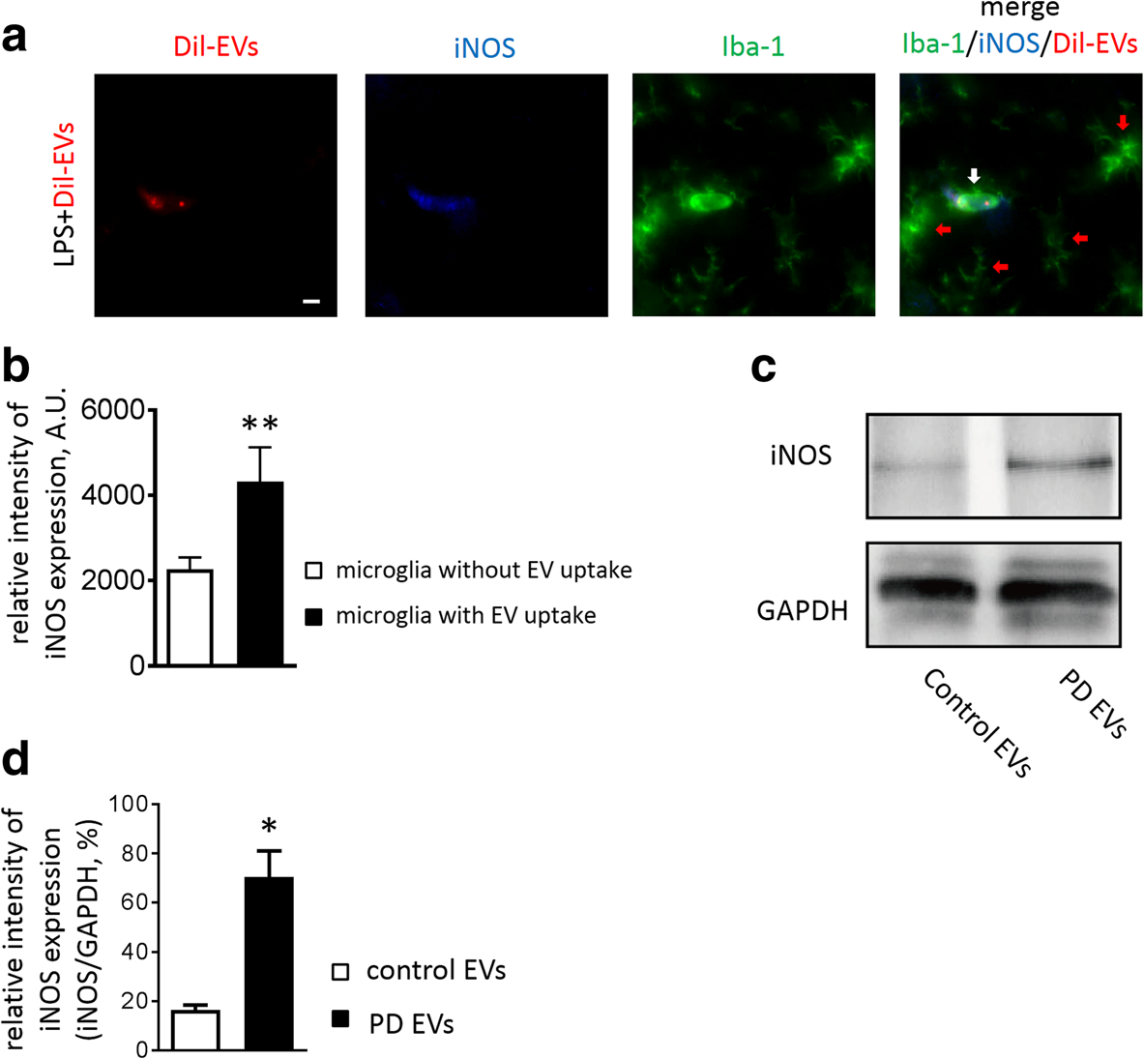
PD-derived EVs exerted a pro-inflammatory effect on microglia with a higher level of iNOS expression compared with healthy control-derived EVs. Representative immunofluorescence images of brain from LPS-treated mice with injection of DiI-EVs co-stained with Iba-1(for microglia) and iNOS. White arrow indicates an iNOS/Iba-1 positive cell with DiI-EVs. Red arrows indicate Iba-1 positive cells without DiI-EVs. Scale bar, 10 μm (**a**) Quantitative analysis of iNOS intesnsity in Iba + microglia in absence or presence of DiI-EVs **b** Values are means ± S.E.M. Student's t-test ** *p* < 0.01 vs microglia without DiI-EVs uptake, *n* = 3 independent animals. Western blot showed PD-EVs induced more iNOS expression compared with normal control-EVs in N9 microglia (**c**, **d**) Values are means ± S.E.M. Student's t-test* *p* < 0.05 vs control-EVs, *n* = 3 independent PD patients and 3 control subjects

Since microglia regulate CNS homeostasis, and activation of microglia promotes CNS inflammation, often with enhanced neuronal degeneration [18], we next investigated whether microglia that have taken up RBC-EVs have an increased expression of the pro-inflammatory factor iNOS. We analyzed the effects of RBC-EVs on microglia in the brains of mice that had been injected systemically with LPS. Brain slices were co-labeled with microglial marker protein Iba-1 and pro-inflammatory factor maker iNOS. We observed the DiI-labeled RBC-EVs were co-localized with iNOS and Iba-1-labeled microglia in the brain. The intensity of iNOS expression showed ~40% increase in microglia containing RBC-EVs when compared to microglia without RBC-EVs (Fig. 5a and b).

To investigate whether RBC-EVs from PD patients can further facilitate inflammation in the CNS, we tested the effect of RBC-EVs from individuals with PD and RBC-EVs from control individuals on iNOS protein and mRNA expression in N9 microglia. Western blot analysis also showed a significant increase in the expression levels of iNOS protein in N9 microglia treated with PD-derived RBC-EVs when compared to microglia treated with control RBC-EVs (Fig. 5c and d).

## Discussion

In this study, we made several major advances with regard to RBC-derived EVs. First, we demonstrate that human RBC-derived EVs, like RBC cell lysate, contain abundant α-syn. Second, we show that these RBC-derived EVs can be transported from peripheral blood into the brain, likely via an adsorptive-mediated transcytosis dependent process across the BBB, in a mouse model with peripheral pre-administration of LPS to induce BBB permeability. This indicates that systemic inflammation, a frequent scenario that occurs in most if not all human subjects at some point, may promote the influx of abundant α-syn-containing RBC-EVs across the BBB. More interestingly, we reveal that this influx resulted in a selective uptake of RBC-EVs by microglia, provoking an increase in microglial inflammatory responses that often lead to enhanced neurodegeneration. RBC-EVs derived from PD patients elicited a stronger microglial inflammatory response than those from control individuals, suggesting that inherent differences in these peripheral vesicles can induce differential effects under conditions of increased influx. We believe our study, for the first time, demonstrates that RBC-derived EVs as a blood-derived source of α-syn can cross the BBB under physiologically plausible circumstances to evoke PD-relevant consequences in the brain.

RBCs have been reported to release EVs, and we were able to obtain purified samples of RBC-EVs by culturing them, and applying the medium to a combination of ultracentrifuge and size exclusion chromatography. Measurement of these particles using NTA revealed that they fall within the range of both exosomes and microvesicles, and further, they expressed markers for RBCs (CD235a) and exosomes (Alix). These data indicate that RBC-EVs obtained by our method are heterogeneous, and likely contain both exosomes and microvesicles. Although RBCs had previously been reported to contain substantial concentrations of α-syn [7], we believe our study is the first to report the abundant expression of α-syn in RBC-EVs (Fig. 1f). Although further detailed characteristics of the EV population based on the size of particle, marker for EVs and their cargo such as α-syn will be necessary to fully understand the individual roles of different sub-types of EVs, our results thus suggest that EVs secreted by RBCs might be important in transporting α-syn as cargo.

In combination with previous studies suggesting important roles for peripheral α-syn in PD (see below), this raised the important question of whether α-syn contained within RBC-EVs can enter and influence the brain. However, little information regarding the distribution and entry of endogenous blood-derived EVs, nor their circulation between blood and brain, is currently available. Our data show that the BBB is largely impermeable to RBC-EVs under healthy conditions. Instead, EVs are accumulated in liver, spleen and kidney, in agreement with other studies of intravenously injected EVs [29, 53].

That said, an inflammatory state may compromise the BBB allowing EV transport from the peripheral circulation to the brain [45]. Our data recapitulate this finding using RBC-EVs, suggesting that endogenous, α-syn-containing EVs are likely to enter the brain during inflammation. Importantly, as shown by capillary depletion (Fig. 2d), these vesicles are not merely captured within the brain microvasculature, but actually enter the brain parenchyma. The relevance of these findings to PD pathogenesis is supported by evidence linking systemic inflammation to PD. People infected with Japanese encephalitis virus and H5N1 influenza virus presented a higher risk for developing PD [27, 51]. In addition, meta-analysis demonstrated elevated peripheral concentrations of cytokines/chemokines in patients with PD [41]. These studies suggest systemic inflammation contributes to development of PD, possibly by propagating pathological signals from periphery to CNS. LPS is a potent activator of immune cells in periphery and CNS, increasing the various pro-inflammatory mediators including cytokines/chemokines and reactive oxygen species (ROS). LPS does not directly affect neurons due to their lack of functional TLR4 expression [16]. Moreover, injection of LPS has proven a useful PD model by which inflammation mediates the progression and selective degeneration of dopaminergic neuron in rodents [16]. Qin L et al. reported that chronic microglia activation and the loss of dopaminergic neurons were observed after LPS-induced systemic inflammation, in a PD model triggered by a single peripheral LPS injection. This report suggested that LPS-induced systemic inflammation triggered the progression of PD [40]. In this study, we observed increased permeability of RBC-EVs containing α-syn under LPS-induced systemic inflammation. However, we have not directly confirmed that transmission of RBC-EVs contributes to the development of PD; therefore, further investigations are needed.

In addition to increasing the influx of RBC-EVs in vivo, treatment with LPS also increased the permeability of RBC-EVs in BMEC monolayer in cell culture (Fig. 2f), suggesting that the mechanism of increased influx may be via an action of LPS on BMECs. Increased influx across the BBB can occur via several mechanisms, including either active transcellular transport or passive paracellular diffusion. Notably, for large particles such as EVs, a “leaky” BBB that allows greater paracellular diffusion may or may not result in greater influx, as these particles typically require an endocytosis-mediated transcellular mechanism and the permeability of the EVs is unlikely simply correlated with the integrity of tight junctions between brain endothelial cells [12]. While our in vitro experiments (Fig. 2e and f) could certainly be consistent with generally increased permeability to all molecules, the in vivo data in Fig. 2 suggests this may not be the case in the animal, as the EV transport is significantly different from the non-specific leakage of albumin, a far smaller substance than an EV. Although we did not explore the mechanism in detail in this work, previous studies have garnered clues to likely candidate pathways. Because clathrin- and caveolae-mediated endocytosis are well known to be major routes of EV endocytosis [31], they present plausible routes for RBC-EVs to enter BMECs, for subsequent export into the brain. LPS induces the endocytosis of vascular endothelial cadherin (VE-cadherin) via clathrin- and caveolae- mediated endocytosis in human vascular endothelial cell line CRL-2922 [61], suggesting that these systems may be increased in our model. Further, LPS is recognized by Toll like receptor 4 (TLR4) [54], which is expressed on the plasma membrane of BMECs, and LPS-TLR4-Src signaling is involved in caveolae-mediated endocytosis of VE-cadherin [61]. Therefore, whether LPS induced direct activation of clathrin- and caveolae-mediated endocytosis, leading to enhanced RBC-EVs permeability through these endocytotic routes, should be investigated. Another important consideration is the effect of inflammatory signaling in general on the permeability of the BBB. As described above, an in vitro study showed that TNF-α increased crossing of HEK293 derived exosomes in an in vitro BBB model and that the crossing of exosomes were mediated by multiple transcytosis routes, not by a paracellular route under exposure of TNF-α [12], and BMECs have been reported to increase AMT in response to TNF-α and IL-6 [14]. Given that LPS induces release of proinflammatory cytokines including TNF-α from BMECs, this exposure may subsequently lead to further enhanced crossing of RBC-EVs at BBB. A related observation is that a single systemic injection of LPS can lead to PD-like pathology featuring microglial activation and dopaminergic degeneration in animals [40].

Taken together, vesicle-mediated transcytosis is an important mechanism by which large molecules cross the BBB, and likely plays a role in the transport of RBC-EVs. Vesicle mediated-transcytosis mechanisms are largely categorized into receptor-mediated transcytosis (RMT) and adsorptive-mediated transcytosis (AMT) [14]. For RMT, some ligands such as transferrin selectively bind to their receptors to cross the BBB [33]; in contrast, AMT is mediated by the adsorption of cationic moieties of particles to anionic components of the plasma membrane [21]. While both mechanisms are saturable, AMT has a lower affinity and higher capacity than RMT [21]. Therefore, we tested whether an LPS-mediated increase in RBC-EVs influx into the brain was characteristic of a high- or low-capacity pathway. Co-injection of ^125^I labeled RBC-EVs with unlabeled RBC-EVs in control mice and LPS mice did not alter the LPS-induced influx, regardless of whether the low (1 μg/mouse) or high (30 μg/mouse) dose of unlabeled RBC-EVs was used. This implication of a non-saturable and/or high-capacity mechanism led us to further probe whether RBC-EVs entered the brain via AMT, using WGA, a lectin that binds to the plasma membrane and increases AMT [4, 59]. As shown in Fig. 3c, WGA increased the uptake of RBC-EVs by brain, particularly in LPS-treated animals. These data showed that RBC-EVs transport at BBB is mediated by AMT, rather than RMT. Further investigations of the process by which RBC-EVs enter the brain remain to be performed, but a particular mechanism of interest is that mediated by heparin sulfate proteoglycans (HSPGs). These proteoglycans are found on the surface of many cell types, leading negative charges on the luminal membrane of endothelial cells, and are involved with mediation of EV uptake in several cells types [13, 15]. Thus, this pathway deserves further consideration. An important caveat, however, is that both the experiments utilizing ^125^I (which labels membrane proteins on the outer portion of the EV membrane), and DiI (which incorporates with the membrane itself) indicate that only a particular fraction of the EV successfully entered the brain parenchyma, and do not prove definitively that intact EVs are present in the brain unchanged. However, that both signals enter the brain signifies that multiple components of the EVs cross, and may remain intact.

Following transfer of EVs across the BBB, we observed RBC-EVs in cerebral nuclei (including striatum), cortex, interbrain (including thalamus and hypothalamus), midbrain and substantia nigra, but not in the hippocampus of LPS injected mice (Fig. 4b). Banks WA et al. showed peripheral injection of LPS increased the BBB permeability of ^14^C-sucrose, which is a small molecule (an indicator of increased permeability via the paracellular route) in cortex, striatum, hippocampus, and midbrain but not in the hypothalamus [2]. Although the BBB integrity to EVs in different brain regions after LPS treatment and the potential selective uptake by region-specific microglia need to be further investigated, the fact that the effect of LPS on the BBB permeability of ^14^C-sucrose partially overlaps with the effect of LPS on the BBB permeability of RBC-EVs suggests that transport of RBC-EVs is not simply due to decrease of integrity of BBB, but mediated by other mechanisms, such as AMT. Regardless of the pathway by which RBC-EVs enter the brain, the consequences of their presence on brain cells must be determined.

Here, we show that RBC-EVs co-localize with brain microglia, in agreement with previous observations that EVs are mainly taken up by microglia [17, 19, 62]. As the resident immune cells of the CNS, activation of microglia induces chronic neuroinflammation, an important feature of PD [38]. Activated microglia release cytokines, reactive oxygen species (ROS) and nitric oxygen (NO). The level of iNOS expression is low in the brain under physiological, non-inflammatory conditions. Increased iNOS expression in the brain was observed in PD patients and PD mouse model [25, 43]. Once expressed, iNOS produces high levels of NO continuously, which is toxic to neurons in PD [43]. In this study, we used the level of iNOS expression as indicator of microglial activation. Interestingly, our data showed that uptake of RBC-EVs provokes an increase of expression of pro-inflammatory iNOS in microglia (Fig. 5a and b). Moreover, EVs derived from the erythrocytes of PD patients promote a greater microglial inflammatory response than those derived from control patients (Fig. 5c and d). Although the human sample number is small in the current study and the results need to be further validated, the significant effects with three replications suggest that EVs arising in the periphery of PD carry inherent differences that may exacerbate the inflammatory effects of RBC-EVs in general. The mechanism of this greater effect of PD EVs was not directly tested, but a previous study demonstrated that the ratio of α-syn oligomer to total RBC protein was higher in PD patients than in control [60], and α-syn is an obvious candidate for the mediating factor in increased inflammatory effects of PD-derived RBC-EVs. It is therefore possible that pathological α-syn protein, possibly in oligomeric form, is carried from blood to brain in EVs under systemic inflammatory conditions. Further investigations are needed to understand whether and how RBC-EVs can carry pathological forms of α-syn and contribute to the loss of dopaminergic neurons in PD. Use of a human α-syn-specific antibody to stain these cells may be useful in answering this question, though attaining sufficient sensitivity to label the likely very low levels of α-syn in individual EVs may be challenging.

A particularly important feature of this microglial response to RBC-EVs is that it may lead to the initiation of a chronic state of inflammation in the CNS, after an event such as sepsis. As discussed above, permeability of BMECs is increased by exposure to pro-inflammatory molecules. Activation of microglia leads to a release of cytokines and reactive oxygen species (ROS), which result in neuronal death [35, 39, 48]. Moreover, these cytokines will interact with the cells of the BBB, potentially prolonging increased BBB permeability, which would, in turn, allow continuing influx of α-syn-carrying RBC-EVs. Thus, a single incidence of increased permeability, caused by an initiating incident of systemic inflammation (e.g., sepsis), could be sufficient to start a cascade of chronic CNS and BBB pathology, particularly in cases in which the RBC-EVs are of enhanced inflammatory potential, such as those observed in PD patients here. Notably, morphological changes RBCs have also been observed in PD patients [37], meaning that the differences in RBC-EVs derived from PD patients may reflect PD-related changes in the erythrocytes themselves. This hypothesis becomes more relevant in considering the pathogenesis of PD in the context of increasing recent evidence for the role of peripheral α-syn in PD. For example, it has been proven possible for pathogenic forms of α-syn to spread from the periphery to the brain via vagal [55, 56], enteric [36], olfactory [44], and intramuscular [46] pathways. Additionally, we have also shown previously that free α-syn in the blood can cross the BBB under both healthy and inflammatory conditions [52], although whether pathological forms (e.g., oligomeric) can do the same is less clear. However, the findings reported here reflect an additional pathway by which α-syn in the periphery may influence the CNS. The potential for such an event to act as one of several synergistic components leading to disease pathogenesis is consistent with the current concept of PD as a disease caused by a complex set of interacting genetic and environmental contributors. Its role in the “prion-like” spread of α-syn pathology remains to be determined, but is a question of great interest.

## Conclusions

In summary, our observation that transport of α-syn-containing RBC-EVs across the BBB under peripheral LPS administration induces microglial inflammatory responses, suggests that RBC-EVs can be an important component in promoting the process of synucleinopathy pathogenesis. A number of questions remain, such as the role and fate of EV-contained α-syn protein in PD pathogenesis. Moreover, whether the systems examined here might be targeted either for therapeutics to prevent or modify the disease, will be a question to further probe as the role becomes clearer.

## Additional files

Additional file 1: Table S1. Demographic and clinical data for control and PD subjects. (DOCX 15 kb)

Additional file 2: Figure S1. Unidirectional influx rates (μL/g-min) for liver, kidney and spleen were calculated by Multiple-time regression analysis, *n* = 14-15/group (A, C, E). Unidirectional influx rates of ^125^I-EVs and Tc^99^m-Alb by liver, kidney and spleen following LPS (B, D, F) Multiple-time regression analysis was used to calculated unidirectional influx rates of Delta values (tissue/serum ratios for ^125^I-EVs with tissue/serum ratios for Tc^99^m-Alb subtracted). (TIFF 943 kb)

## Abbreviations

BBB: Blood-brain barrier
BMECs: Brain microvascular endothelial cells
CNS: Central nerve system
CSF: Cerebrospinal fluid
EVs: Extracellular vesicles
expt: Exposure time
IL: Interleukin
iNOS: Induced nitric oxide synthase
LPS: Lipopolysaccharide
PD: Parkinson’s disease
RBCs: Red blood cells
TLR4: Toll like receptor 4
TNF-α: Tumor necrosis factor-α
α-syn: Alpha-synuclein
