# Supplementary material for: Transmission of α-synuclein-containing erythrocyte-derived extracellular vesicles across the blood-brain barrier via adsorptive mediated transcytosis: another mechanism for initiation and progression of Parkinson’s disease?
Source: Acta Neuropathol Commun. 2017 Sep 13;5:71. doi: 10.1186/s40478-017-0470-4 (PMC5598000; doi:10.1186/s40478-017-0470-4)
Supplement: Supplementary file 1 — Demographic and clinical data for control and PD subjects. (DOCX 15 kb) [file 40478_2017_470_MOESM1_ESM.docx]

| **Subject** | **Age** | **Gender** | **Duration/year** | **UPDRS** | **H-Y** | **Onset** | **Medication beginning** | **Medication** |
| --- | --- | --- | --- | --- | --- | --- | --- | --- |
| **PD** | **67** | **male** | **8** | **36** | **2.5** | **2008** | **2008** | **Trastal**  **Madopar** |
| **PD** | **52** | **female** | **1** | **12** | **1** | **2016** | **-** | **-** |
| **PD** | **84** | **Male** | **1** | **33** | **2** | **2016** | **2016** | **Pramipexole**  **Madopar** |
| **HC** | **72** | **male** | **-** | **-** | **-** | **-** | **-** | **-** |
| **HC** | **86** | **female** | **-** | **-** | **-** | **-** | **-** | **-** |
| **HC** | **60** | **Male** | **-** | **-** | **-** | **-** | **-** | **-** |
