# Supplementary figures and images for: Transmission of α-synuclein-containing erythrocyte-derived extracellular vesicles across the blood-brain barrier via adsorptive mediated transcytosis: another mechanism for initiation and progression of Parkinson’s disease?
Source: Acta Neuropathol Commun. 2017 Sep 13;5:71. doi: 10.1186/s40478-017-0470-4 (PMC5598000; doi:10.1186/s40478-017-0470-4)

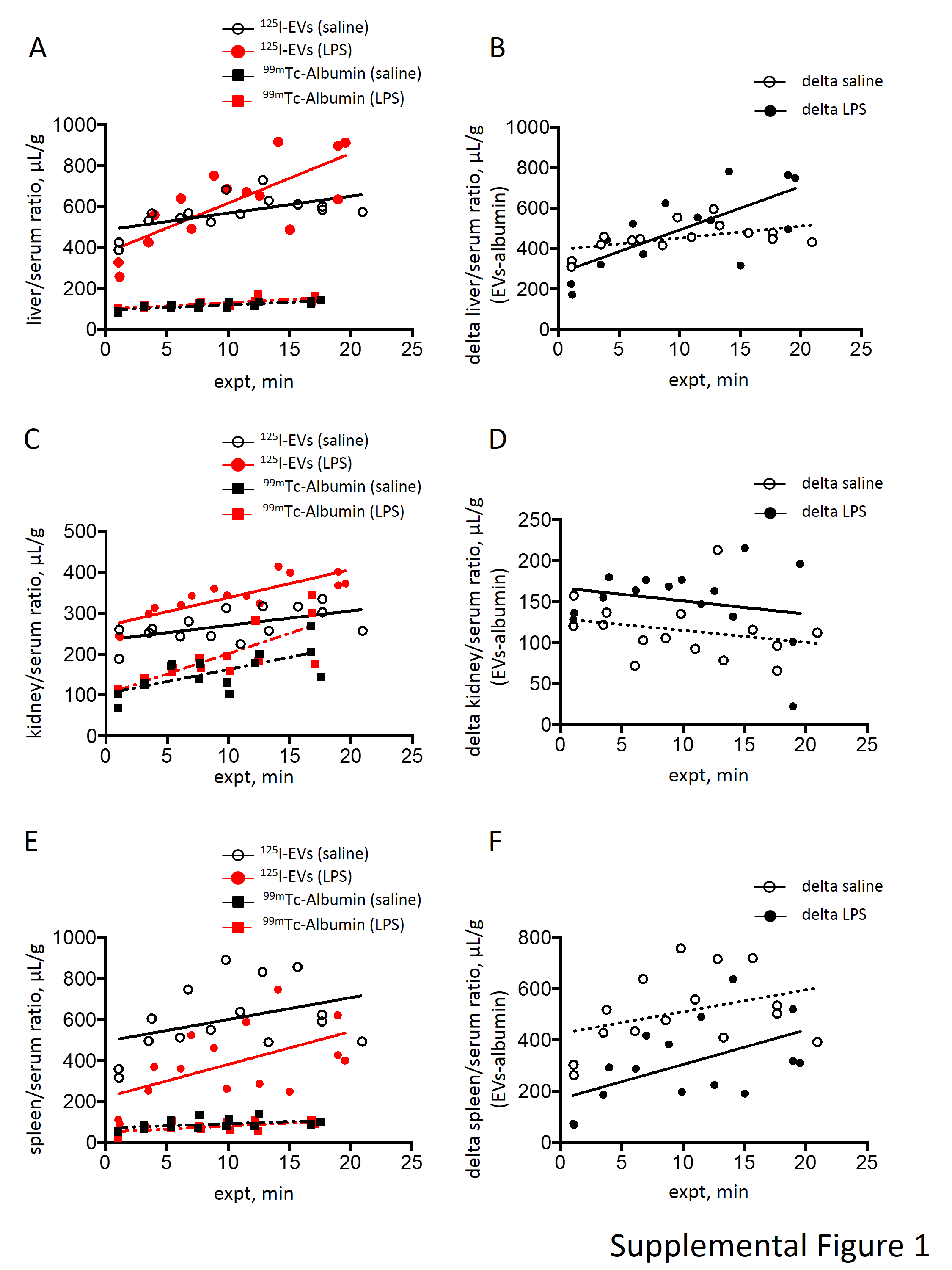

Supplement: Supplementary file 2 — Unidirectional influx rates (μL/g-min) for liver, kidney and spleen were calculated by Multiple-time regression analysis, n = 14-15/group (A, C, E). Unidirectional influx rates of 125I-EVs and Tc99m-Alb by liver, kidney and spleen following LPS (B, D, F) Multiple-time regression analysis was used to calculated unidirectional influx rates of Delta values (tissue/serum ratios for 125I-EVs with tissue/serum ratios for Tc99m-Alb subtracted). (TIFF 943 kb) [file 40478_2017_470_MOESM2_ESM.tif]
